# Nursing in the ICU: comparison of the NAS and time on bedside

**DOI:** 10.1186/cc10205

**Published:** 2011-06-22

**Authors:** DF Moura, NM Lucinio, A Pardini, S Shiramizo, MR Ribas, RA Morbek, ELPCA Rosa, MAA Yamashita, MR Guerra, E Silva

**Affiliations:** 1 Hospital Israelita Albert Einstein, São Paulo - SP, Brazil

## Introduction

The increasing costs of treatment in intensive care units (ICUs) and the need to use resources efficiently require adequacy between nursing staff and nursing workload, as a high cost is attributed to the nurse staff of ICUs. The intensity of the nursing work effort should be considered because staffing needs vary according to the amount of patients being cared for, as well as the type of care provided for each of those patients. As the intensity of the nursing work effort increases, the amount of nursing staff required to properly care for patients also increases.

## Objective

To analyze the adequacy of nursing staff according to NAS, and compare the time of care according to NAS and time of care according to Nurse Call.

## Methods

An exploratory, descriptive prospective study was performed in an adult 32-bed ICU of a private general hospital in São Paulo, Brazil. In our study we included 18 beds for which the Nurse Call System by Austco was available. The Nurse Call System by Austco enables nurses to provide prompt and effective responses to patients' calls at all times. For the analysis of the adequacy of the nursing staff, the mean NAS expressed as percentage time was initially converted into hours considering a 6-hour shift (6 hours equivalent to an NAS of 100%).

## Results

Follow-up of 1,710 patients who were admitted to the ICU between July and December 2009 resulted in 4,592 NAS assessments. Analysis of the nursing workload showed a mean NAS of 90.1 ± 4.4% (ranging between 82.9 and 93.7%). The number of patients ranged from 26.5 to 34.7 in the ICU. The ICU occupation rate fluctuated between 82.8 and 113.9%, during the study, suggesting that managing of the unit was suboptimal. The hours available for nursing care in the 6-hour shift remained constant throughout the studied period and represented a total of 156 hours per shift-day. This number was the same for the entire study period, as the number professionals was fixed. According to the NAS, during half of the studied period (July to September) there was a need for an increased number of nursing professionals, as there was an average deficit of 30 hours (range 4.4 and 48.9 hours). In the second half of the study (October to December) the number of nurses available exceeded that considered necessary by NAS. This surplus was of 14.2 hours on average (range 9.0 and 22.5). The time required for nurse care per patient per day was very similar between the two assessment tools (NAS and Nurse Call). While for NAS the mean time required by patient was 5.4 hours per day (ranging between 5.0 and 5.6), for the Nurse Call this time was 5.3 hours per day (ranging between 4.9 and 5.5).

## Conclusion

The Nurse Call System can help the ICU nurse manager on the staff required, showing us a new strategy for managing the nurse staff. Regarding it being more easy to use, it can be adequately evaluated in the ICU.

